# Stützverbände: Eigenschaften von Materialien und biomechanische Eigenschaften

**DOI:** 10.1007/s00064-025-00895-9

**Published:** 2025-05-14

**Authors:** Klaus Dresing, Theddy F. Slongo

**Affiliations:** 1 Klinik für Unfallchirurgie, Orthopädie und Plastische Chirurgie, Universitätsmedizin Göttingen, Robert-Koch-Str. 40, 37075 Göttingen, Deutschland; 2 Childrens University Hospital, Dept. of Paediatric Surgery, University of Bern, Freiburgstrasse 20, 3010 Bern, Schweiz

**Keywords:** Gipsverbände, Schienen, Semirigide Materialien, Rigide Materialien, Biomechanische Prinzipien, Surgical casts, Splints, Semi-rigid materials, Rigid materials, Biomechanical principles

## Abstract

Stützverbände dienen der Ruhigstellung und Immobilisation von Gliedmaßen nach jeglicher Art von Verletzungen, die einer entsprechenden Immobilisation bedürfen. Je nach der Art der Verletzung kommen verschiedene Arten von Stützverbänden sowie auch verschiedene Materialien zum Einsatz. Aufgrund der zunehmend operativen Behandlung von Frakturen im Kindesalter beobachten wir einen rasanter Verlust der „handwerklichen“ Fähigkeit zur optimalen Applikation besonders von Gipsverbänden. Im Nachfolgenden sollen einerseits die verschiedenen Materialien von Stützverbänden und deren Eigenschaften dargestellt und andererseits auch die entsprechenden Indikationen aufgeführt werden.

## Abstract (en)

Support bandages and casts are used to immobilize and stabilize limbs after various types of injuries that require appropriate immobilization. Depending on the type of injury, different types of support bandages/casts and different materials are used. Due to the increasing surgical treatment of fractures in children, we are observing a decline in the practical skills needed to properly apply supportive bandages, particularly plaster casts. In the following, the different materials of support bandages and their properties are presented as well as their corresponding indications.

## Definition und Geschichte von Stützverbänden

Als Stützverbände werden sämtliche immobilisierende Verbände bezeichnet. Hierzu zählen Gipsverbände und -schienen, Immobilisationen mit Kunststoffmaterialien und auch jegliche Art von Orthesen. In diesem Beitrag soll ein kurzer Überblick über Stützverbandmaterialien, den prinzipiellen Aufbau und Komplikationsvermeidung gegeben werden.

Bereits bei ägyptischen Mumien konnte die Frakturbehandlung im Papyrus Smith – nach dem Tod von Smith übersetzt vom Ägyptologen James Henry Breasted – (2494–2345 v. Chr.) nachgewiesen werden [8, 11]. Hippokrates beschrieb 350 v. Chr. Schienungen von Frakturen, bei denen Holzstäbe mit Bandagen fixiert und mittels Wachs versteift wurden [4]. Der Niederländer Mathijsen streute Gips in Textilgewebe und gilt damit als Entdecker der Gipsruhigstellung [7]. Lorenz Böhler, Wien, perfektionierte die konservative Knochenbruchbehandlung Anfang des 20. Jahrhunderts [2]. In den 1950er-Jahren kamen synthetische Materialien in die Klinik [1, 12, 14]. In den 1970er-Jahren führte Sarmiento die Brace-Behandlung ein [9, 10].

## Gipsraum

### Ausstattung

Im Gipsraum sollten eine gepolsterte Liege und Lagerungsmaterial wie Kissen, Rollen etc. vorhanden sein. Instrumente zur Stützverbandanlage und Bearbeitung sind vorzuhalten. Für die Bildgebung sind ein Röntgenbildwandler (C-Bogen) und – wenn immer möglich – ein Sonographiegerät im Raum vorhanden [4]. Bei Weißgipsverarbeitung sollte der Abfluss mit Gipsabscheider ausgestattet sein, um ein Verstopfen der Abflüsse zu verhindern. Weißgipsmaterial muss trocken gelagert werden.

### Instrumente

Standardmäßig sind folgende Instrumente bzw. Geräte im Einsatz:

- oszillierende Säge (Akku empfehlenswert),
- Lister-Verbandsschere,
- Bruns-Schere,
- Universalschere,
- Stille-Gipsschere,
- Cast-Spreizer,
- Cast-Biege-Zange,
- Gipsmesser,
- Gipssäge.

**Abb. 1:**
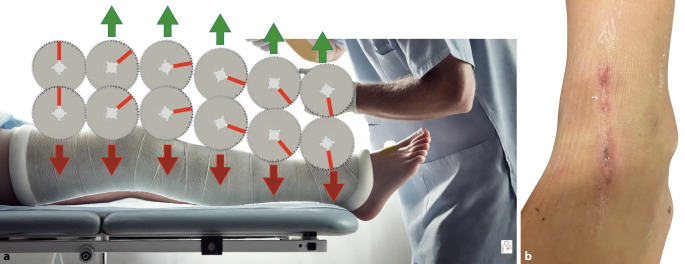
**a** Korrekte Nutzung der Gipssäge: Um Hitzeschäden an der Haut zu vermeiden, sollte das oszillierende Sägeblatt in den Verband kurz eingetaucht und nach dem Herausziehen etwas gedreht werden, bevor es wieder in den Stützverband geführt wird. Damit werden Überhitzungen des Sägeblatts gemindert. **b** Verletzung der Haut durch Gipssäge: Narbe nach insuffizienter Anwendung der oszillierenden Gipssäge

Die oszillierende Säge führt häufig beim Patienten zu Angst, hier ist Aufklärung vorbeugend. Die Lautstärke wird als unangenehm empfunden. Durch Hitzeentwicklung und falsche Anwendung kann es zur Verletzung der Haut kommen. Zur Vermeidung von Komplikationen hat sich bewährt [5], die Säge mit Finger am Stützverband abzustützen und die Sägeblattposition ständig zu ändern (bzw. zu drehen; [4, 13]; Abb. 1).

## Stützverbandmaterialien

Bei den Stützverbandmaterialien unterscheiden wir Weißgips (Plaster of Paris [POP]), synthetische Materialien, Bandagen, Tapes und die verschiedenen Polstermaterialien (Tab. 1).

**Tab. 1:** Vergleich der Eigenschaften von synthetischem Stützverbandmaterial und Weißgips. (Mod. nach [4])

Eigenschaft	POP/Gips	Synthetisches Material	
		Rigide	Semirigide
Wasserfest	↓	↑↑↑	↑↑↑
Perspiration, Durchlässigkeit	↑	↑↑	↑↑
Hautirritation bei Anlage	↑	↑↑	↑↑
Sofortige Belastbarkeit	↓	↑↑	↑↑
Modellierbarkeit	↑↑↑	↑	↑
Dicke/Größe/Gewicht	↑↑	↓	↓
Druckresistenz	↓	↑↑	↑
Kompressionswiderstand	↑↑	↑↑↑	↑
Akutversorgung	↑↑↑	↑	↑↑
Unterstützung Reposition	↑↑↑	↑	↑
Funktionelle Stabilisierung	↑	↑↑	↑↑↑
Relation Stärke zu Gewicht	↑↑	↑↑↑	↑↑/↑↑↑
Primäre definitive Versorgung	↑	↑	↑↑↑

*POP* Plaster of Paris

Erklärung der Eigenschaften: ↑↑↑ gut/hoch, ↑↑ moderat, ↑geeignet, ↓nicht geeignet

### Weißgips

Weißgips oder allgemein Gips ist Calciumsulfatdihydrat (CaSO_4_-2H_2_O). Das Gipspulver in der Binde (Calciumsulfathemihydrat) + Wasser (H_2_O) bilden unter exothermer Reaktion wieder CaSO_4_-2H_2_O.

**Abb. 2:**
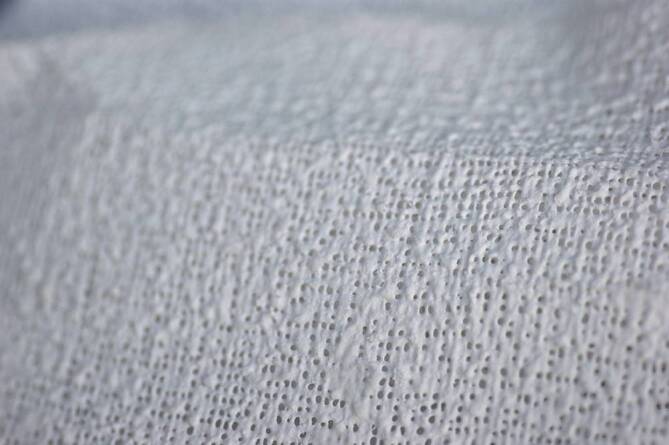
Detailaufnahme Gipsbinde: In das Textilgewebe ist Gips eingestreut und mit einem Bindemittel fixiert

**Abb. 3:**
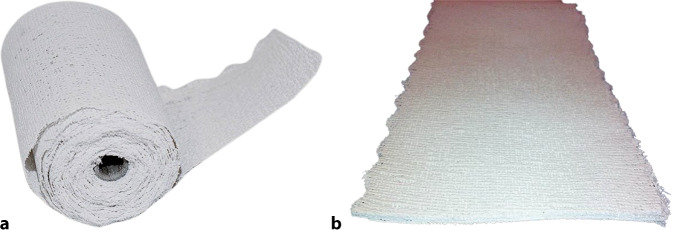
**a** Gipsbinde: Auf einem Konus ist das Gipsmaterial aufgerollt. **b** Gipslonguette: Das Material wird in Bahnen geliefert, Verwendung für Schienen oder in Kombination mit Gipsrollen

Für die Gipsverbände wird Baumwollgewebe verwendet, in das Gipspulver eingestreut ist. Ein Bindemittel verhindert das Herausfallen des Pulvers (Abb. 2). Das Material steht in Form von Rollen oder Longuetten zur Verfügung (Abb. 3).

Das Gipsmaterial ist sehr an die anatomischen Gegebenheiten anpassungsfähig, es ist sehr gut modellierbar. Das Material ist porös und absorbiert Flüssigkeit wie Blut und Sekret.

Das Stützverbandmaterial hat eine hohe Steifigkeit, aber keine Ausdehnfähigkeit. Die Stabilität des Gipsverbandes ist abhängig von der Kristallstruktur, dem unbeeinflussten Aushärtungsprozess, der Anzahl der Lagen und natürlich der Compliance des Patienten.

Um ideale Eigenschaften des Weißgipsstützverbandes zu erzielen, sind folgende Punkte zu beachten: korrekte Verarbeitung mit einer Tauchtiefe 20–30 cm (15–20 °C), einer kompletten Durchnässung des Gipsmaterials ohne trockene Areale und adäquate Lagenzahl des Materials.

**Abb. 4:**
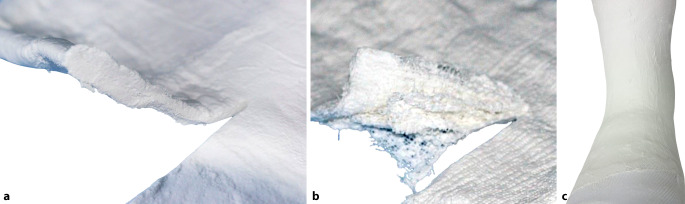
Gipsverarbeitung: **a** gut ineinander gewalkter Gips: Die einzelnen Lagen sind homogen verbunden und bilden eine Schicht. **b** Die einzelnen Gipslagen sind nicht verbunden, Resultat: Blätterteiggips und damit mangelnde Stabilität

Bei der Verwendung von Gipsrollen sollten diese beim Eintauchen in das Wasser schräg gehalten werden, damit Luftblasenaustritt ermöglicht wird. Bei Verwendung von Gipslonguetten müssen die Gipslagen zusammengestrichen, gewalkt werden, damit ein homogener Gips entsteht, sonst resultiert eine Art „Blätterteigeffekt“ ([4]; Abb. 4).

Auf die Temperatur des Wassers ist zu achten, hohe Wassertemperatur kann zu Verbrennungen führen. Die Verarbeitungszeit von Gips beträgt etwa 3–5 min. Die initiale Aushärtung ist nach 10–12 min, die komplette Aushärtung erst nach 24–48 h erreicht.

Für die Spaltung und Entfernung wird die oszillierende Säge eingesetzt.

### Synthetische Materialien

**Abb. 5:**
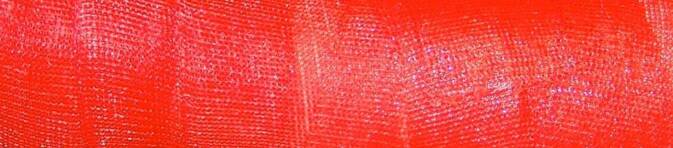
Synthetisches Stützverbandmaterial

Bei synthetischen Stützverbandmaterialien werden Lagen von Polyestergewebe, Fiberglasgewebe, Polypropylengewebe mit einem Präpolymer Polyurethan-Harz getränkt (Abb. 5).

Bei den Materialien unterscheiden wir rigides Material („hard cast“) von semirigidem Material („soft cast“). Diese Materialien sind nur mit Handschuhen zu verarbeiten, weil sonst die Polymerisate an den Fingern kleben bleiben.

**Abb. 6:**
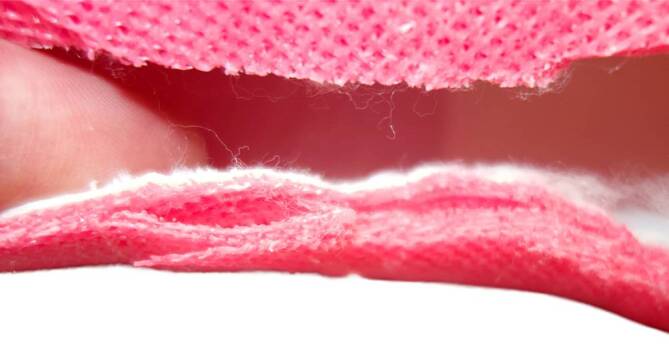
Insuffizienter Lagenverbund bei semirigidem Stützverbandmaterial, Resultat: Blätterteigeffekt mit resultierender mangelnder Stabilität

Durch Kontakt mit Luft, deutlich schneller durch Wasserkontakt polymerisiert das Material zum Polymer aus und stabilisiert. Auch bei diesem Material ist darauf zu achten, dass der Lagenverbund erzielt wird, weil sonst ein Separieren der Materialschicht resultieren kann und mangelnde Stabilität folgt (Abb. 6). Fiberglasfreies Polymer (Thermoplast) kommt ebenfalls zur Anwendung.

#### Rigide Materialien

Rigides Material hat harte Kanten und erfordert gute Polsterung der Stützverbandenden, um Irritationen und Verletzungen der Haut zu vermeiden. Das Material wird mit der Cast-Säge gespalten und bearbeitet.

Das Material ist leicht, haltbar, hat scharfe Kanten, und bei der Anwendung werden Handschuhe getragen, um nicht Reste des Kunststoffs an den Händen nach der Anwendung zu haben.

#### Semirigide Materialien

Semirigide Stützverbandmaterialien sind ebenfalls leicht und sehr haltbar. Sie können mit der Schere gespalten und getrimmt werden Dieses Material eignet sich auch gut für das Containment nach dem Sarmiento-Prinzip. Handschuhe werden bei der Anwendung getragen.

#### Combi-Cast-Technik

**Abb. 7:**
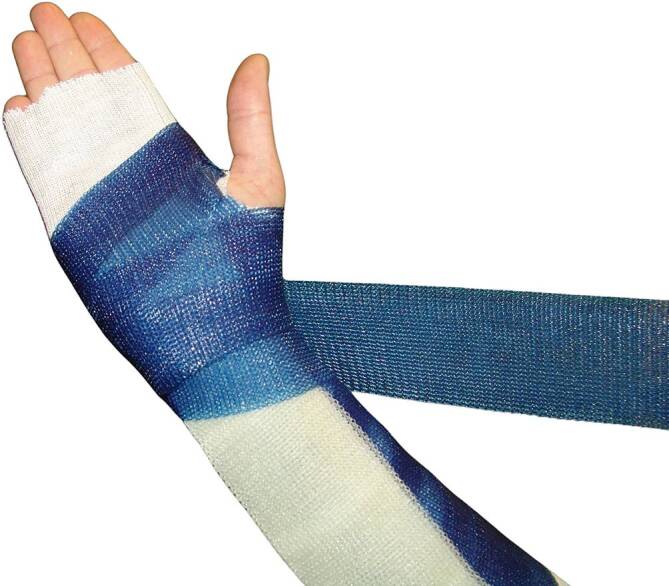
Combi-Cast-Stützverband: In semirigides Material wird eine Hard-cast-Longuette eingelegt. Effekt ist Stabilisierung des Gelenks bei Vorteil des semirigiden Materials

Bei der Combi-Cast-Technik wird in semirigides Material „hard cast“ integriert (Abb. 7).

### Verarbeitungszeit

Synthetische Stützverbandmaterialien haben eine deutlich kürzere Aushärtungszeit als Weißgips. Nach 2–4 min ist der Polymerisierungsprozess weitgehend abgeschlossen. Nach 6–8 min ist der Verband stabil, nach 30 min komplett ausgehärtet.

## Polsterung

Eine Polsterung unter einem Stützverband ist angezeigt zum Schutz der Haut und Weichteilgewebe. Aber es sollte nur so viel Material verwendet werden, wie gerade notwendig ist. Knöcherne Vorsprünge und oberflächliche Nerven sollten sicher und mit genügend Material geschützt werden, um Druck auf Nerven und Gewebe zu verhindern. Folgende Areale sollten beachtet werden: Akromion, Skapula, laterale und mediale Humeruskondylen, Olekranon, Processus styloideus radii und ulnae, Trochanter major, Femurkondylen lateral und medial, Patella, ganz besonders Fibulaköpfchen, Tibiakante, Außen- und Innenknöchel, Kleinzehe, proximale Interphalangealgelenke (PIP), Großzehe, Symphyse, Steißbein, Processus spinosi, Sakrum, Spinae iliacae anterior und superior.

Polsterung verhindert auch direkte Reibung zwischen Stützverbandmaterial und Haut und kann Exkoriationen der Haut und damit Geschwürbildung verringern. Außerdem kann Polsterung zur Vermeidung thermischer Verletzungen während der Aushärtung beitragen.

### Untermaterial

**Abb. 8:**
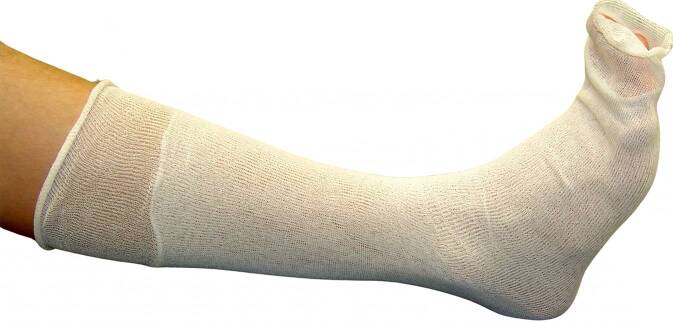
Unterbau: erste Lage des Stützverbands mit Baumwollschlauch/Schlauchverband als Hautschutz

Normalerweise wird ein Strumpf, Schlauchverband, Stockinette aus Baumwolle oder aus Mischgewebe mit Viskose und elastischen Fasern als erste Lage direkt auf die Haut gebracht (Abb. 8). Dieses Material ist weich, aber nicht sehr formbar, es kann zu Falten führen, und das Material kann auch reißen. Es ist nicht wasserdicht.

### Polsterwatte

**Abb. 9:**
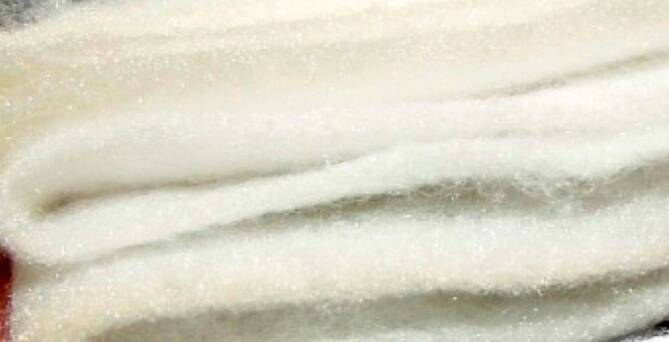
Synthetische Polsterwatte

Über diese erste Schicht kann Polsterwatte, meist aus Mischgewebe (Viskose und elastische Fasern), gelegt werden (Abb. 9). Dieses Material ist weich, nicht saugfähig, aber meist hoch atmungsaktiv und formbar.

### Synthetisches Polstermaterial

**Abb. 10:**
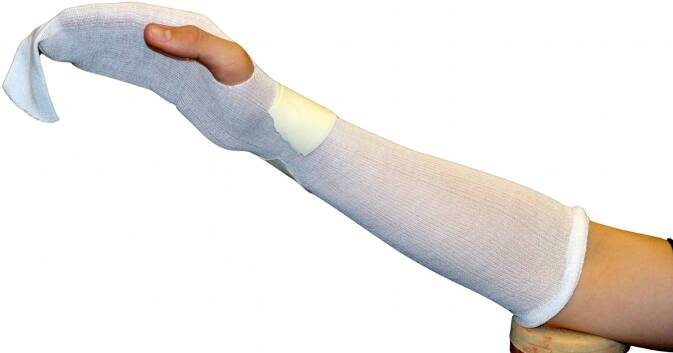
Unterbau: Polsterung der prominenten Areale mit Schaumstoffklebematerial (Foam) auf dem Schlauchverband, hier Processus styloideus radii und ulnae

Hier kommt offenporiges Schaumstoffmaterial, meist selbstklebend, zum Einsatz, oder adhäsives Polstertape wird verwendet (Abb. 10). Polstertape kann direkt auf die erste Lage des Schlauchverbandes aufgeklebt werden.

## Arten von Stützverbänden

Grundsätzlich sollten alle Rollen/Binden so gehalten werden, dass die Rolle in der Hohlhand liegt oder zwischen Daumen und Zeigefinger gehalten wird. Die überlappende Applikation aller Materialien wird empfohlen [4].

Bei der Applikation von Stützverbänden sollte mit Ausnahmen die Funktionsstellung der Gelenke eingehalten werden, um durch die Ruhigstellung nicht Sehnenverkürzungen zu erzeugen [4]. Auf eine korrekte Länge aller Stützverbände sollte geachtet werden, um funktionelle Behandlungen und Beübungen der freien Gelenke zu ermöglichen.

Bei frischen Verletzungen sollten die Stützverbände bis auf die letzte Faser gespalten werden, um der posttraumatischen Schwellung Ausdehnungsmöglichkeit zu geben [2].

Bei allen Stützverbänden sollte am Ende die Durchblutung z. B. durch Nachweis der Kapillardurchblutung überprüft und dokumentiert werden [4]. Instruktionen sind allen Patienten zu geben und zu dokumentieren.

### Schienen

**Abb. 11:**
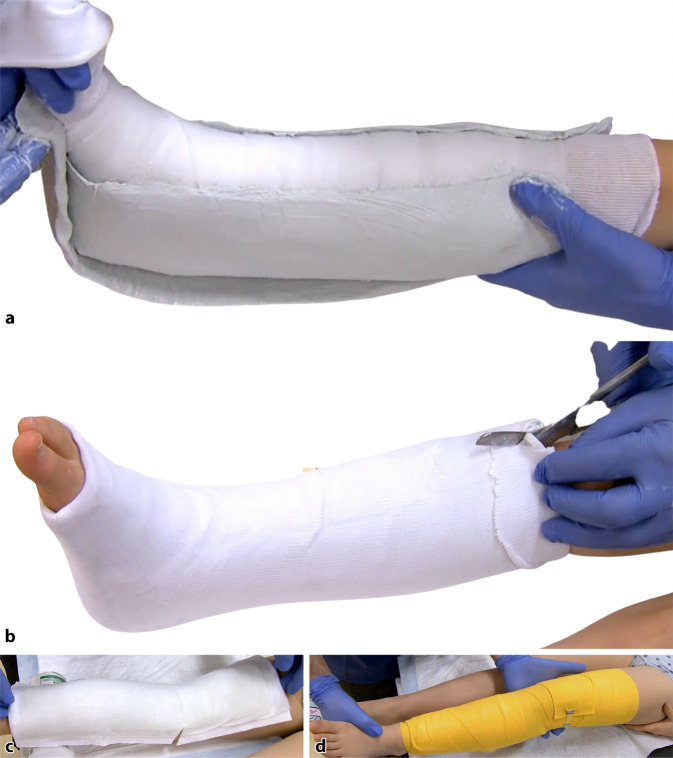
Stützverbandschienen: **a** U-L-Gipsschiene am Unterschenkel. **b** In der Primärversorgung Spaltung des Verbandes bis auf die letzte Faser, um Schwellungsausdehnung der Weichteile zu ermöglichen. **c** Kunststofffertigschiene gepolstert. **d** Fixierung mit elastischer Binde

Ist eine vollständige Ruhigstellung nicht erforderlich, z. B. nur um Schwellungen zu verringern oder um weitere Weichteilschäden zu vermeiden, kann eine nichtzirkuläre Schiene aus Gips oder Kunststoff angelegt werden. Bei der Erstversorgung von Frakturen und Gelenkverletzungen in der Notaufnahme werden solche nichtzirkulären Gipsverbände in der Regel angelegt, da im Verlauf der Behandlung akute und sekundäre Schwellungen auftreten können. Eine weitere Indikation für das Anlegen einer Schiene ist die postoperative Behandlung oder als Schutz nach einer Osteosynthese, um sicherzustellen, dass der Verband nicht mit der postoperativen Schwellung in Konflikt gerät. Verschiedenste Anwendungen sind beschrieben (Abb. 11).

Sowohl Gips als auch synthetische Materialien können zur primären, temporären, sekundären oder definitiven Behandlung von akuten Frakturen, Verstauchungen oder Zerrungen eingesetzt werden. Zur Ruhigstellung von entzündeten Weichteilen und Sehnen, Extremitäten oder Gelenken können einfache Längsschienen verwendet werden. Zur Ruhigstellung der oberen Extremität, des Unterarms oder bei Verletzungen des Sprunggelenks werden Schienen mit U‑förmiger Technik (bei der das Material auf einer Seite der Gliedmaße nach unten und auf der anderen Seite wieder nach oben aufgebracht wird) oder Böhler-U-Schienen verwendet.

Vor dem Anlegen einer Schiene wird ein Schlauchverband oder ein Strumpf über die Extremität gezogen und die entsprechende Polsterung angebracht. Es ist wichtig zu wissen, dass eine zu starke Polsterung die immobilisierende Wirkung der Schiene beeinträchtigt, da zwischen den verschiedenen Schichten des Verbandes und der Haut zu viel Platz ist. Lorenz Böhler bevorzugte einen nahezu ungepolsterten, aber gut geformten Gips [2]. Er verwendete die Polsterung nur zum Schutz von Knochenvorsprüngen.

Bei der Verwendung von Gips oder synthetischem Material hängt es von dem Ort der Verletzung und der Kraft des Patienten ab, wie viele Lagen der Polsterung verwendet werden sollten.

Um die Stabilität zu erhöhen, sollte die Schiene etwa zwei Drittel des Umfangs der Extremität abdecken. Eine weitere Möglichkeit ist die Verwendung kleiner Streifen des Stützverbandmaterials zur Verstärkung der Stabilität in Bereichen, in denen mehr Stabilität erforderlich ist, z. B. um Gelenke herum.

Bei der Verwendung von Gips besteht eine Schiene für die obere Extremität in der Regel aus 8 bis 10 Lagen, für die untere Extremität werden 12 bis 16 Lagen benötigt. Da die Steifigkeit von synthetischem Material größer ist, werden für die obere Extremität nur 6 bis 8 Lagen benötigt, für die untere Extremität reichen 9 bis 12 Lagen aus.

### Rundverbände/zirkuläre Stützverbände

Ein zirkulärer Stützverband wird immer dann eingesetzt, wenn ein höheres Maß an Ruhigstellung erforderlich ist, oder bei der Sekundärbehandlung. Die Stabilität eines vollständig zirkulären Stützverbandes ist wesentlich höher als die einer Schiene. Ein zirkulärer Stützverband erlaubt die Belastung und das Gehen, was mit einer Schiene oder einem gespaltenen Gips nicht möglich ist.

Um einen Gipsverband so stabil zu machen, dass er belastet werden kann, kann es erforderlich sein, die zirkulären Wicklungen mit zusätzlichen Streifen oder Longuetten zu kombinieren, um die Bereiche des Gipsverbandes zu verstärken, in denen er leicht brechen könnte, insbesondere in der Nähe von Gelenken.

Bei einem zirkulären Stützverband wird die Extremität mit einer Schlauchbinde (Stockinette) geschützt und die erforderliche Polsterung angebracht.

Das Stützverbandmaterial (Rolle) wird von distal nach proximal um die Gliedmaße gewickelt. Beim Wickeln sollte das Cast-Material mindestens 50 % überlappen (Halbüberlappungstechnik), um Schwachstellen zu vermeiden. In Bereichen, in denen die maximale Belastung der Extremität auftritt, sind mehr Wicklungen erforderlich. Für einen zirkulären Stützverband sind 8 bis 10 Lagen Weißgips oder 4 bis 6 Lagen Kunststoffmaterial erforderlich, um eine ausreichende Stabilität für die obere Extremität zu erreichen. Für Gipsverbände der unteren Extremitäten werden 12 bis 14 Lagen Gips oder 6 bis 8 Lagen synthetisches Material empfohlen.

Alle zirkulären Gipsverbände, die zur *Primärbehandlung* (erster Verband nach einer Verletzung oder einem chirurgischen Eingriff) angelegt werden, müssen vollständig gespalten und mit einem elastischen Verband oder einer kohäsiven Binde gesichert werden. Andernfalls wird die postoperative Weichteilschwellung durch den Verband eingeschränkt, was zu einer Kompression der Weichteile führt. Dies kann ein Kompartmentsyndrom verursachen. Da einige Stützverbandmaterialien und Verbände beim Trocknen schrumpfen können, sollte der Arzt den Stützverband nicht nur spalten und lockern, sondern auch alle darunter liegenden Polsterschichten nach der ersten Aushärtung das Stützverbandmaterial durchtrennen.

### Gespaltene Verbände

**Abb. 12:**
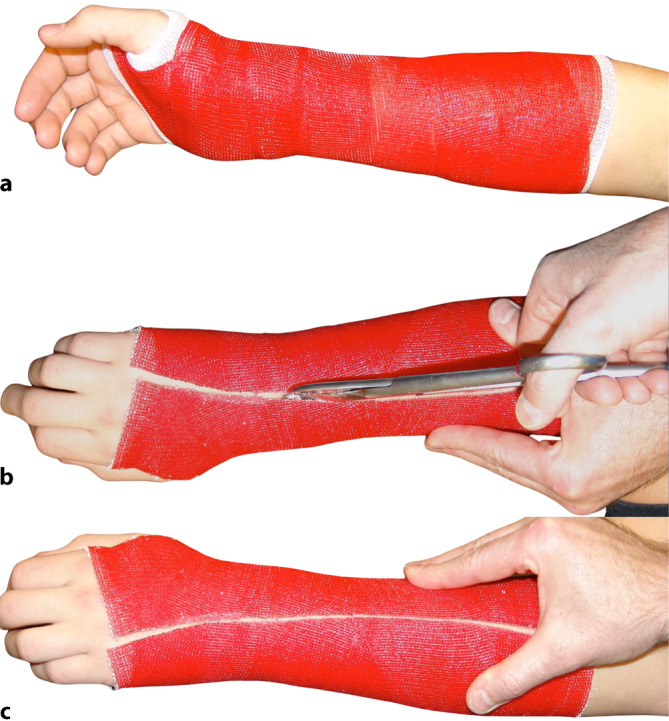
**a** Primärer Unterarmstützverband aus semirigidem Material. **b** Spalten mit der Schere bis auf die letzte Faser am Unfalltag. Nach Abschwellung kann auch ein schmaler Streifen herausgeschnitten werden, um einen festen Sitz und effektive Stützfunktion wieder zu erzielen. **c** Nach Enfernung des Streifens kann der Verband geschlossen und mit einer Haft- oder elastischen Binde refixiert werden

Wird ein zirkulärer Stützverband bei einer frischen Fraktur oder postoperativ angelegt, sollte er gespalten werden (in Längsrichtung), um eine posttraumatische oder postoperative Schwellung zu ermöglichen, ohne den Gewebedruck zu erhöhen (Abb. 12**)**. Wird der Verand nich gespalten, können Komplikationen auftreten, wie z. B. eine Beeinträchtigung der venösen oder arteriellen Durchblutung, Nervenreizungen oder Kompartmentsyndrome mit potenziell dauerhaften Verletzungen der Weichteile, insbesondere der Muskeln und Nerven.

Ein Gips oder Hard-Cast wird mit einer Gipssäge, ein semirigider Stützverband mit einer Schere vollständig gespalten und ggf. mit einem Gipsspreizer geweitet. Eine andere Technik besteht darin, einen 1 cm breiten Streifen zu entfernen, um mehr Platz zu schaffen. Diese Lücke wird mit einer Polsterung aufgefüllt und der gespaltene Gips mit einer elastischen Binde umwickelt, um eine Schwellung entlang der Lücke (Spaltödem) zu verhindern.

### Orthesen

**Abb. 13:**
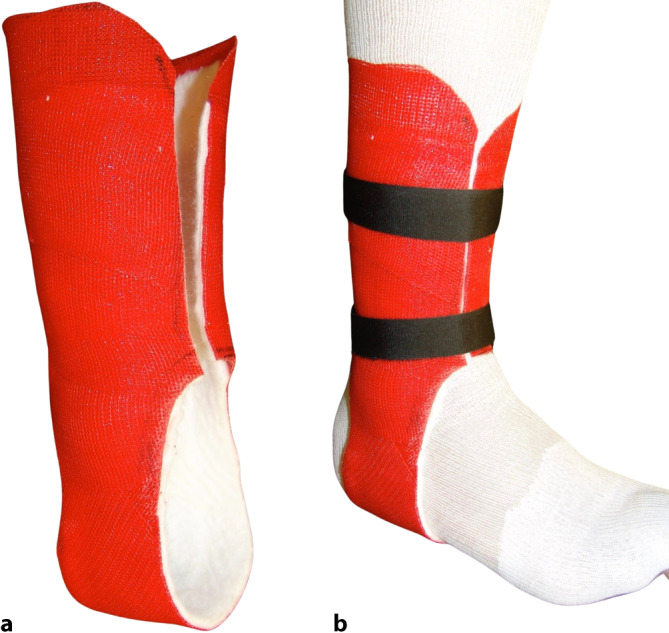
**a** Zugeschnittene Sprunggelenkorthese. **b** Orthese am Patienten mit Klettband fixiert

Orthesen sind abnehmbare Stützverbände, die individuell gefertigt angepasst werden oder vorgefertigt und anpassbar sind. Eine „Orthese“ ist eine abnehmbare externe orthopädische Vorrichtung (Abb. 13), die die Bewegung von Gliedmaßen, Kopf oder Wirbelsäule verhindert oder kontrolliert. Während Gipsverbände die Domäne des Chirurgen und des Gipstechnikers sind, werden Orthesen in der Regel von anderen Fachkräften des Gesundheitswesens wie Orthopädietechnikern und Ergotherapeuten angelegt, und die Vorrichtungen selbst sind in der Regel leicht zu entfernen und wieder anzulegen, auch vom Patienten selbst. Beispiele für Orthesen sind die Halo-Weste oder Halsmanschetten für den Kopf- und Nackenbereich oder Gurtbandagen für den Arm oder das Fußgelenk. Zu den wichtigsten Arten von Orthesen gehören:

- kommerziell erhältliche, vorgefertigte Geräte. Sie sind unterschiedlich gut an den Patienten anpassbar. Die Technik besteht darin, die angefertigten Vorrichtungen aus Leder oder Kunststoff, Metall und anderen Komponenten individuell anzupassen. Hierzu werden teilweise vorher Abdrücke der Gliedmaßen angefertigt.
- Vorrichtungen aus thermoplastischem Material, das speziell an die Konturen der Gliedmaße des Patienten angepasst ist,
- abnehmbare Stützverbände und Schienen aus synthetischen Gussmaterialien, die speziell für jeden Patienten entwickelt werden.

Am besten wird eine Orthese nach Abklingen der posttraumatischen Schwellung angelegt. Wenn eine Frakturorthese so konstruiert ist, dass der Patient den Sitz der Orthese anpassen kann, um sie optimal zu stützen, ist sie noch anpassungsfähiger. Eine vorübergehende Entfernung der Frakturschiene kann auch für einige physiotherapeutische Behandlungen oder die Körperhygiene hilfreich sein.

## Biomechanische Prinzipien

Bei einer Fraktur wird das mechanische und muskuläre Gleichgewicht gestört. Es kommt zur Dislokation der Fraktur durch das Unfallereignis direkt oder Muskelzug. Nach der Reposition einer Fraktur müssen diese genannten Kräfte aufgefangen werden. Ein Stützverband dient also als eine äußere Stabilisierung einer Fraktur.

Das erforderliche Maß an Gegenkraft hängt von der Höhe der auf die Extremität einwirkenden Kraft ab. Bei einer Unterarmfraktur muss ein Gips oder eine Schiene oberhalb des Ellenbogens angelegt werden, da sonst die auf die Fragmente wirkenden Kräfte die Gegenkraft des Stützverbandes übersteigen würden. Bei einem Knöchelbruch hingegen ist der Abstand zu proximaler Tibia und Fibula so groß, dass ein Stützverband unterhalb des Knies ausreicht, um die auf die Fragmente wirkenden Kräfte auszugleichen. Das Drei-Punkte-Prinzip muss nicht beachtet werden, da die entstehenden Kräfte durch die proximale Tibia oder Fibula ausgeglichen werden. Es sollte immer angestrebt werden, so viel Bewegung wie möglich zuzulassen, und wenn keine Notwendigkeit besteht, das proximale Gelenk zu immobilisieren, sollte dies nicht getan werden.

### Drei-Punkte-Prinzip

Das klassische mechanische Prinzip aller Stützverbände ist die Dreipunktabstützung nach Charnley [3].

**Abb. 14:**
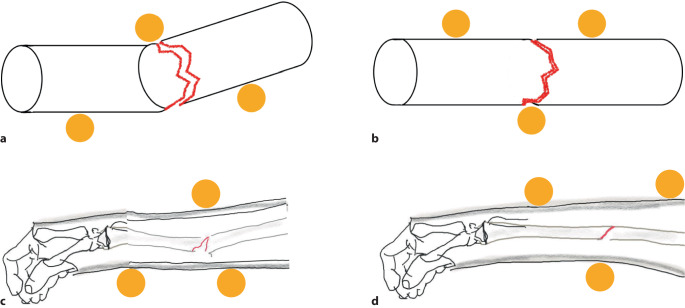
Prinzip der Dreipunktabstützung. **a** Schematisch nach Unfall. **b** Reposition und Dreipunktabstützung im Stützverband. **c** Krafteinwirkung bei Fraktur am Beispiel einer Radiusschaftfraktur. **d** Nach Reposition und Gipsanlage

Die Stabilisierung von nur 2 Punkten distal und proximal der Fraktur reicht fast nie aus, um die Angulationskräfte zu kontrollieren. Es wird ein dritter Punkt benötigt, um der Kraft entgegenzuwirken (Abb. 14).

Die Dreipunktabstützung ist für die Neutralisation der Kräfte bei der Immobilisation und Stabilisierung erforderlich.

Das Drei-Punkte-Prinzip muss bei einer Sprunggelenkfraktur nicht beachtet werden, da die entstehenden Kräfte durch die proximale Tibia oder Fibula ausgeglichen werden. Es sollte immer angestrebt werden, so viel Bewegung wie möglich zuzulassen, und wenn keine Notwendigkeit besteht, das proximale Gelenk zu immobilisieren, sollte dies nicht getan werden. Allerdings kann bei einem zu kurzen Stützverband am Unterschenkel der Stabilisierungseffekt verloren gehen.

### Hülsenfunktion/Brace/Prinzip PET-Flasche gefüllt

Ein weiteres Prinzip der Frakturstabilisierung ist der Hydraulikeffekt der Weichteile in einer Stützverbandhülse. Sarmiento konnte zeigen, dass Weichteil- und Muskeldruck um eine Frakturregion herum Fragmente reponieren oder zumindest ins Alignement bringen können, wenn die Hülse den Muskeldruck auf den Knochen zurückgibt [6, 9, 10].

**Abb. 15:**
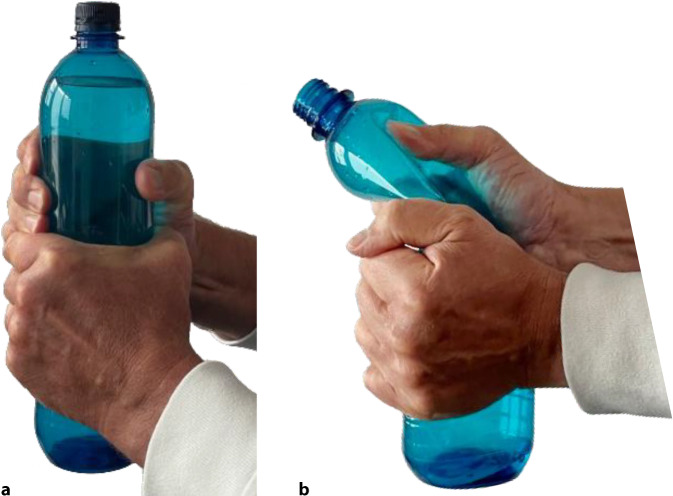
Demonstration des hydraulischen Prinzips nach Sarmiento am Beispiel einer Wasser-PET-Flasche. **a** Gefüllt lässt sich die Flasche nicht komprimieren, entsprechend agieren die Muskeln in einer Stützverbandhülse. **b** Ohne Wasser kollabiert das System, die Flasche lässt sich zusammenfalten

Bei dieser Methode werden die angrenzenden Gelenke nicht immobilisiert, und durch die funktionelle Behandlung und Beübung werden Muskelatrophien weitgehend vermieden. Am besten lässt sich diese Technik mit einer wassergefüllten PET-Flasche verdeutlichen. Bei gefüllter und verschlossener Flasche lässt sich die Flasche nicht komprimieren. Die leere PET-Flasche kann leicht zusammengefaltet werden (Abb. 15**)**.

## Aufklärung

In Abhängigkeit von Notfallsituationen vs. selektiver Applikation ist immer eine ausführliche Aufklärung erforderlich [4]. Folgende Punkte sollten abgehandelt werden: alternative Behandlungsoptionen, Haut‑, Weichteil‑, Nerven‑, Gefäßirritation, Stützverbanddruckstellen (Nervendruck, Hautnekrosen), allergische Reaktion auf das Stützverbandmaterial, Gipssägeverletzungen, Redislokation der Fraktur, Stützverbandbruch. Die Patienten sollten aufgeklärt werden, wann eine Wiedervorstellung notwendig ist: bei zunehmenden Schmerzen, starker Schwellung, zunehmendem Juckreiz, Hautirritation, neurologischen Ausfällen: sensible, motorische, Bewegungeinschränkungen, Bewegungsdefizite angrenzender Gelenke, Blasenbildung, Lockerung oder Bruch des Stützverbands.

## Probleme und Komplikationen

Werden zu viele Schichten Polstermaterial verwendet, ist der Schienungseffekt des Stützverbandes vermindert, insbesondere nach Abschwellung. Deshalb sollte nach dem Prinzip so wenig wie nötig, so viel wie erforderlich gehandelt werden.

Falten im Polstermaterial können zu Druckstellen führen und müssen vermeiden werden.

**Abb. 16:**
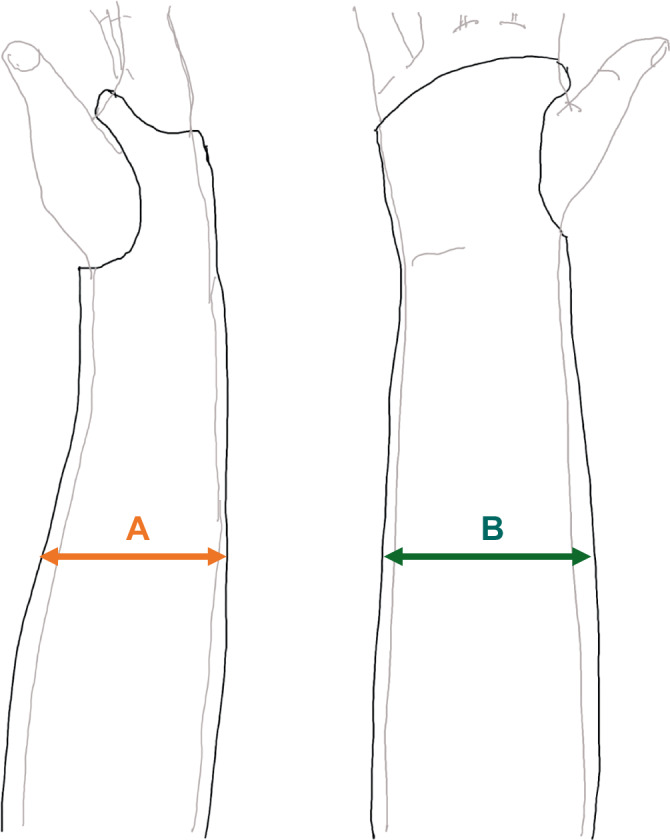
Cast-Index: sagittaler Stützverbandinnendurchmesser/koronarer Durchmesser

Ein Röntgenbild eines gut angelegten Unterarm-Casts zeigt einen Gipsindex von 0,7 oder weniger (Innenbreite in der Seitenansicht (sagittal)/Breite in der a.p.-Ansicht (coronar); CI [Cast-Index] = A/B; Abb. 16). Mit dem Cast-Index können einfach Redislokationen bei Unterarmfrakturen von Kindern im Stützverband vorausgesagt werden.

Die korrekt angelegte Dreipunktformung ist wichtig, um Redislokationen zu vermeiden.

**Abb. 17:**
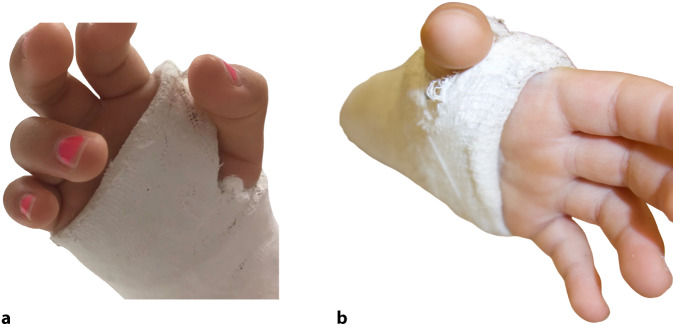
**a** Distal wird die Handgelenkbeugefalte nicht respektiert, der Verband ist zu weit nach distal und der Stützverband ist im 1. Interdigitalraum zu weit: Weder die Finger nach der Daumen können adäquat bewegt werden. **b** Der Steg zwischen Daumen und Zeigefinger ist zu breit und behindert die Daumenbewegung

Beim Anlegen der Stützverbände ist auf die korrekte Länge zu achten. Gelenke, die nicht ruhiggestellt werden sollen, müssen frei beweglich ohne Behinderung durch den Stützverband sein. So sollte der Unterarmstützverband die Ellenbeugung nicht behindern. Distal sollte die Daumenbeweglichkeit nicht eingeschränkt sein (Abb. 17).

## Fazit für die Praxis

- Stützverbände dienen der Ruhigstellung und Immobilisation von Gliedmaßen nach jeglicher Art von Verletzungen, die einer entsprechenden Immobilisation bedürfen.
- Je nach Verletzung kommen verschiedene Arten von Stützverbänden sowie auch verschiedene Materialien zum Einsatz. Bei den Stützverbandmaterialien werden Weißgips, synthetische Materialien, Bandagen, Tapes und die verschiedenen Polstermaterialien unterschieden. Daneben kommen auch Orthesen als abnehmbare Stützverbände zum Einsatz.
- Das klassische mechanische Prinzip aller Stützverbände ist die Dreipunktabstützung nach Charnley.
- Der Patient sollte über die Risiken und Komplikationen der Behandlung aufgeklärt werden, sofern eine Notfallsituation dies nicht verhindert.
